# The Attallah screw: Where safety meets robustness in posterior subaxial cervical instrumentation

**DOI:** 10.3892/mi.2024.159

**Published:** 2024-04-24

**Authors:** Mohammed Hasanain, Colya N. Englisch, Thomas Tschernig, Samah Saeed, Magomed Lepschokov, Ralf Ketter, Joachim Oertel

**Affiliations:** 1 Department of Neurosurgery, Saarland University Medical Center, D-66421 Homburg/Saar, Germany; 2 Institute for Anatomy and Cell Biology, Saarland University, D-66421 Homburg/Saar, Germany

**Keywords:** spine surgery, subaxial cervical spine, lateral mass, pedicle, Attallah screw, peak torque

## Abstract

Posterior fixation of the subaxial cervical spine (SCS) commonly relies on the application of lateral mass screws (LMS), with pedicle screws being a less prevalent alternative. The present study provides another option: A recently introduced novel approach, the Attallah screw, intended to ensure a safety profile comparable to that of LMS, combined with a strength profile similar to that of pedicle screws. The focus of the present study is the comparative analysis of peak insertion torques for these three screw types. Employing standard surgical techniques and instruments, Attallah screws were scheduled for insertion on the right side of the SCS in 15 cadavers, pedicle screws on the left side in 8 cadavers, and LMS on the left side in the remaining 7 cadavers. The peak insertion torque was recorded using an electronic torque screwdriver. The results revealed that the peak insertion torques were similar in the pedicle and the Attallah screw at C3, C4 and C7, but differed at C5 (mean ± SD; pedicle, 79.5±19.6 cNm; Attallah, 56.7±18.5 cNm; P=0.029) and C6 (pedicle, 85.4±28.7 cNm; Attallah, 49.8±17.9 cNm; P=0.004) in favor of the superior pedicle screw measurements. The peak insertion torques of the pedicle screw were superior to the corresponding data from the LMS from C4 to C7. By contrast, the peak insertion torques of the Attallah screw were only superior to those of the LMS at C7 (Attallah, 69.5±24.5 cNm; lateral mass, 40.5±21.4 cNm; P=0.030), although similar trends were observed at the other cervical levels. On the whole, the findings presented herein indicate the level-dependent superior robustness of the Attallah screw as a posterior cervical fixation method compared to the LMS. However, from a biomechanical perspective, the pedicle screw remains the preeminent choice for fixation within the C5-C6 range.

## Introduction

Posterior cervical spine instrumentation is frequently indicated to treat subaxial cervical spine (SCS) instability. Degeneration, tumors, infections, and trauma are common etiological factors (1). The posterior fixation of the SCS essentially relies on lateral mass screws (LMS), whereas pedicle screws are less popular due to initial studies reporting the concomitant increased risk of neurovascular injury (e.g., spinal cord, nerve roots, and vertebral arteries) (2-8). Even though the risk of violation of the pedicle wall has been reported to reach up to 27.9% (9), both spinal cord injuries and vertebral artery lesions remain relatively rare (10-12). Recent meta-analyses do not corroborate such a high occurrence of neurovascular injuries indeed (13,14). On the other hand, in view of the anatomical dimensions of the lateral mass and the pedicle (15-19), LMS are substantially restricted in length and robustness compared to pedicle analogues (15,20). As a compromise between safety (e.g., neural and arterial injury) and insertable screw length, the authors of the present study have previously suggested the use of the unique dimensions and safe placement of the posterior part of the transverse process (19,21) as a new target for posterior fixation along the SCS (19). In a follow-up study, the comparability of the posterior part of the transverse process and the subaxial cervical pedicle with respect to the maximum insertable screw length was established (20). This new so-called Attallah approach of screw placement along the most lateral part of the lamina towards the transverse process has already been incorporated with extremely satisfying clinical results (unpublished data).

Thus, the aim of the present cadaveric study was to investigate the biomechanical properties measured as insertion peak torque of the three different screw types along the SCS.

## Materials and methods

### Study design

The present study was based on 15 cadavers, 9 males and 6 females. The mean age of the cadavers was 78.8 years, with a standard deviation of ±9 years. All cadavers were from the body donation program of the Institute for Anatomy and Cell Biology of Saarland University, Homburg/Saar, Germany. The subjects had died of natural causes, such as age, infections, or tumors. The present study was approved by the Ethics Committee of the Saarland Medical Association (approval no. 73/21). Patient consent was given for the publication of the computed tomography (CT) scans and for the use of the cadavers in research in general. Prior to the procedure, CT scans of the cervical spine were performed on all cadavers. Cadavers with tumors or extreme deformities, including cervical kyphosis and scoliosis, were excluded from the study. A posterior approach to the cervical spine was implemented, mimicking real-life scenarios, and using the same instruments, until exposure of the posterior cervical spine landmarks was achieved. The Attallah screw (Neon3 System, Ulrich Medical) was foreseen for the insertion on the right side of subaxial vertebrae (C3 to C7) of all 15 cadavers. On the left side, pedicle screws were scheduled for insertion in 8 cadavers, while LMS were scheduled for insertion in 7 cadavers. Screw dimensions are provided in Table I. Cadaveric positioning, draping, and surgical dissection procedures, including the use of standard surgical instruments, were meticulously adhered to in order to mirror established practice in an operating theatre. Following a midline incision, a midline dissection technique involving subperiosteal elevation of the paraspinal muscles was employed for the purpose of localizing both the lateral masses and facet joints bilaterally. The entire cervical spine, spanning from C1 to T1, was methodically exposed, enabling the identification of all relevant anatomical bony structures, including facet joints, lateral masses, and spinous processes. In order to replicate the challenges encountered in the operating room with regard to obtaining the angulations necessary during screw insertion, the paraspinal muscles were not removed. This approach was imperative not only to simulate realistic difficulties but also to prevent a potential bias which could arise from a facilitated determination of the screw trajectory when the entire spine is exposed.

All screws were inserted by an experienced spine surgeon to ensure precision and avoid errors. Consistent with a previous study, the Ulrich Medical Neon3 System was also used in the present study (19,20).

### Instrumentation and measurements

**Figure 1:**
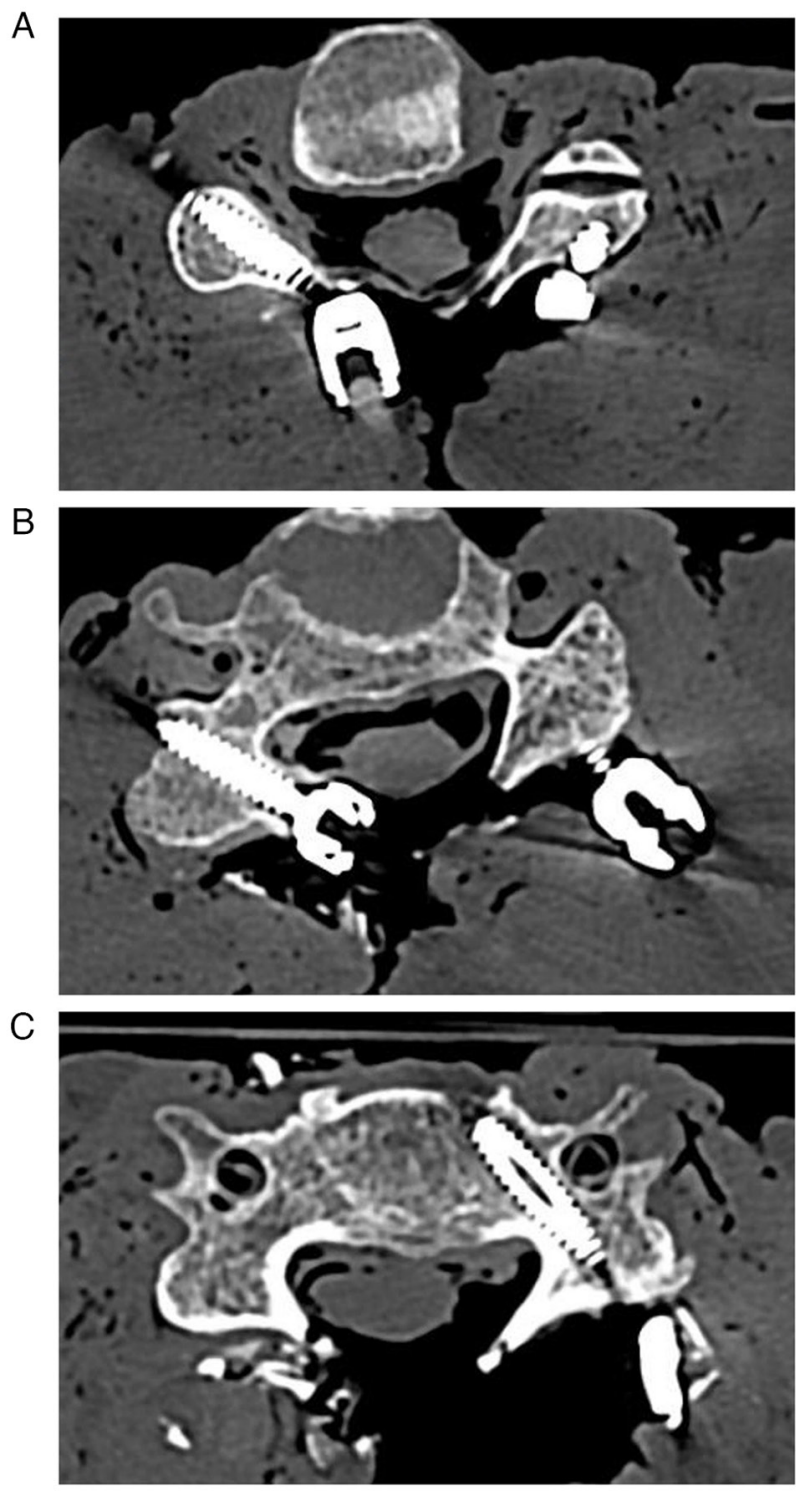
(A) Axial cut CT scan of a cadaver illustrating the placement of an Attallah screw at C4 on the right cadaver side (3.5 mm diameter, 18 mm length) and a lateral mass screw on the left cadaver side (3.5 mm diameter, 12 mm length). (B) Axial cut CT scan depicting the presence of an Attallah screw at C5 on the right cadaver side (3.5 mm diameter, 16 mm length). (C) Axial cut CT scan revealing the placement of a pedicle screw at C5 on the left cadaver side (4.0 mm diameter, 24 mm length). CT, computed tomography.

**Figure 2:**
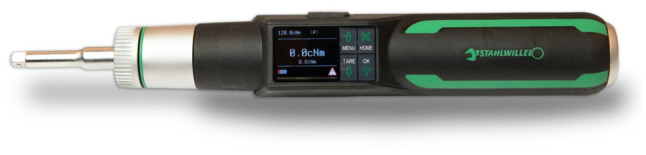
The torque screwdriver STAHLWILLE TORSIOTRONIC^®^. The image is presented with the kind authorization from STAHLWILLE Eduard Wille GmbH & Co. KG.

All three screw types were inserted following the creation of an entry point using a 4 mm diamond burr and a 2 mm high-speed drill to prepare the screw path. The integrity of the screw path was confirmed using a fine probe. The Attallah screw was inserted in accordance with the published trajectory and technique (20). The LMS was inserted using Margel's technique as previously described (22), and the pedicle screw was positioned based on anatomical landmarks. As in standard operating room procedures, laminectomies were conducted in order to further facilitate the insertion of the pedicle screw. A Penfield dissector No. 4 (Aesculap AG) was used to precisely localize the medial wall of the targeted pedicle; an approach which proved effective in attaining positive results. Using a freehand technique without X-ray guidance, the same surgeon inserted all the screws up to 1 mm depth until the screw head came into contact with the underlying bony cortex. In order to ultimately confirm accurate screw positioning and to exclude screw contact with the vertebral artery and the joint space, CT scans were performed on all cadavers (Fig. 1). All scans were reviewed utilizing sagittal and coronal reconstructions in conjunction with the axial cuts based on an evaluation sheet with predetermined criteria (presence of violation of the spinal canal, violation of the facet joint, violation of the intervertebral foramen, violation of the transverse foramen, bony fractures, determination as to whether the screw is bicortical, and evaluation of the accuracy of the planned screw trajectory). The scan reviewer is an experienced senior spine surgeon specialized in complex and deformity spine surgery. Torque measurements during insertion were performed using an electronic torque screwdriver (STAHLWILLE TORSIOTRONIC^®^, STAHLWILLE Eduard Wille GmbH & Co. KG, Wuppertal; Fig. 2). The measuring unit was set in centinewton meter (cNm), and both the reproducibility and accuracy of the measurements were regularly verified. The peak torque of insertion for each screw was measured and recorded.

### Statistical analysis

Statistical analysis was performed using the GraphPad Prism software package (version 10.1.0; Dotmatics). Descriptive statistics were provided using the mean value and the standard deviation (mean ± SD). In view of the yet small sample size, parametric tests were applied. Comparisons among unpaired groups were performed using the ordinary one-way analysis of variance (ANOVA) followed by Tukey's multiple comparisons test. Equal variances among groups were investigated prior to one-way ANOVA using the Brown-Forsythe test. P-values (stated as ‘P’) were two-sided and a value of P<0.05 was considered to indicate a statistically significant difference.

## Results

All screws were placed accurately and safely, and, as confirmed through CT-based verification, no revisions were necessary. Cranial and lateral insertion angulations mimicked the ones used in daily clinical routine and were dependent upon the screw placement type and its spine level (Table II). In one of the cadavers, the cortex of the lateral mass of C3 was cracked on both sides, with the vertebral body remaining intact. This was presumably due to an incidental injury during acquisition of the specimen. To avoid inaccuracy the decision was made to exclude this vertebra from the study. All other C3 specimens were intact and adequate for the study. Thus, the total number of inserted screws was 146, subdivided as follows: Pedicle screws, 38; Attallah screws, 73; LMS, 35.

**Figure 3:**
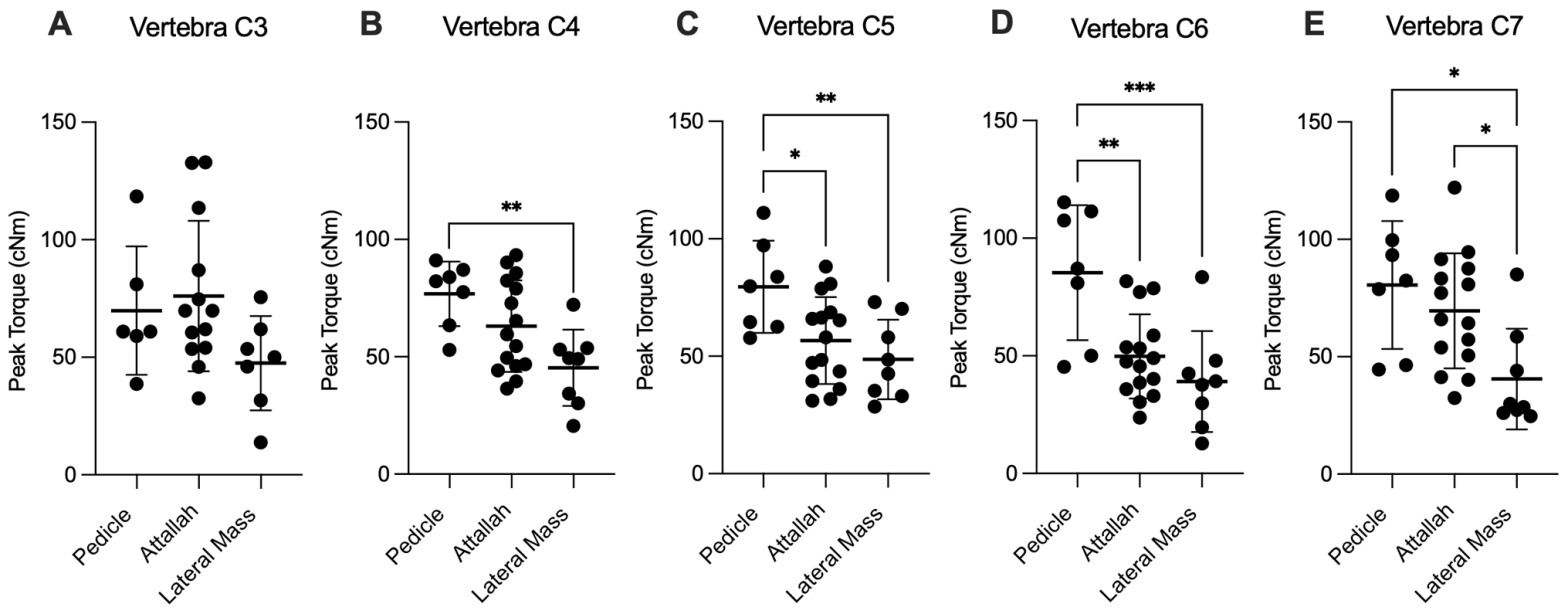
Comparison of the peak insertion torques along the subaxial cervical spine. (A) C3, (B) C4, (C) C5, (D) C6, and (E) C7 in dependency of the employed screw type. Each point represents an individual measurement from an individual cadaver. The horizontal bars represent the mean value with the standard deviation. ^*^P<0.05, ^**^P<0.01 and ^***^P<0.001, determined using one-way ANOVA with Tukey's post hoc test.

Throughout the cervical spine (C3 to C7), no statistically significant differences with respect to intervertebral-level peak torque were detected in any of the three investigated screws. One single exception constituted the Attallah screw measurements between C3 and C6 (P=0.027, Tukey's test). Statistically significant differences in peak torque were observed among the different screw types from C4 to C7 (Fig. 3B-E). No statistically significant difference was, however, observed at the C3 level, although the LMS measurements (47.5±20.1 cNm) appeared to be lower than corresponding data from the pedicle (69.8±27.3 cNm) and the Attallah screw (76.1±32.0 cNm; P=0.117, ANOVA; Fig. 3A). In addition to this, the data from C4 and C7 displayed similar peak torques between the long screws (Fig. 3B and E). In detail, at the C4 vertebral segment, the pedicle screw (76.8±13.8 cNm) peak torque measurements were not statistically superior to those of the Attallah screw (63.0±19.5 cNm) but were instead superior to those of the LMS analogues (45.3±16.3 cNm; P=0.005, Tukey's test; Fig. 3B). Furthermore, at the C7 level, both the pedicle (80.5±27.3 cNm) and the Attallah screw (69.5±24.5 cNm) peak torque values were statistically superior to those from the LMS (40.5±21.4 cNm; P=0.010 and P=0.030, respectively; Tukey's test; Fig. 3E). A different result, involving significant differences between the pedicle and the Attallah screw, emerged at the C5 and C6 vertebral segments (Fig. 3C and D). Namely, at the C5 level, the pedicle screw peak torque values (79.5±19.6 cNm) were statistically significantly superior to the corresponding data from both the Attallah screw (56.7±18.5 cNm; P=0.029, Tukey's test) and the LMS (48.7±16.9 cNm; P=0.008, Tukey's test; Fig. 3C). The respective differences between the pedicle screw (85.4±28.7 cNm) and both the Attallah screw (49.8±17.9 cNm; P=0.004, Tukey's test) and the LMS (39.1±21.5 cNm; P=0.001, Tukey's test) were even more accentuated at the C6 level (Fig. 3D). On the whole, these data indicate biomechanical similarities between the pedicle and the Attallah screws at the C3, C4 and C7 levels, but not at the C5 and C6 levels.

## Discussion

### Biomechanics

The present study determined the insertion peak torques of the pedicle screw, the Attallah screw, and the LMS in the SCS. In brief, peak insertion torques were similar between the pedicle and the Attallah screw at C3, C4 and C7, but differed at C5 and C6. While a statistical comparison of the pedicle screw with the LMS revealed significant differences from C4 to C7, this is less clear when comparing the LMS with the Attallah screw: Although statistical significance could not always be ascertained, there were relevant differences in the mean values at the C3, C4 and C7 levels. At the C5 and C6 levels, the differences in the mean values accounted only for ~10 cNm. This is an interesting observation in view of the fact that these two levels were the only statistically significant ones in the comparison between the pedicle and the Attallah screw. This associates with the Attallah screw length, which is shortest (16 mm) at the C5 and C6 levels. As already mentioned, screw length is largely dependent on the anatomical dimensions (20). Notably, a previous study by the authors demonstrated that the dimensions of the pedicle and the posterior part of the transverse process mostly differed in width precisely at the C5 and C6 levels along the SCS (19). This indicates that not only the length of the anatomical structure is a critical factor when considering robustness, but also its width. The biomechanical benefit which the Attallah screw offers, becomes ultimately clear in view of the fact that pedicle screws are less commonly used in posterior instrumentation of the SCS (e.g. short segment) than the LMS.

### Safety

As already mentioned, LMS is a safe and therefore frequently used tool in posterior subaxial instrumentation (3,20). However, due to the anatomical dimensions of the subaxial cervical lateral mass, the insertable screw length is restricted (15,20). The results of the present study are concomitant with those of previous research, indicating that short screws are limited in robustness compared to longer screws (15). By contrast, the pedicle enables the insertion of long and robust screws (5,15,20). On the other hand, it carries a risk of neural and vascular injury (6,12). To bypass the respective screw disadvantages and to enable safe positioning with longer, and thus potentially robust screws, the present study proposed the site of the posterior part of the transverse process. More precisely: The insertion trajectory that crosses the junction of the lamina with the lateral mass, the pedicle and the posterior part of the transverse process heading towards the posterior tubercle (19,20). Attallah screws can be used very safely and effectively in combination with posterior decompression of the cervical spine. The starting point of the Attallah screw is indeed more medial in comparison to that of the LMS, thus avoiding the extreme lateral dissection needed to insert pedicle screws. It also provides a better area laterally for bone graft placement as, compared to LMS, the head of the screw is more medially located. A clinical study from the authors' department addressing this aspect is upcoming. Moreover, in certain cases, particularly those including instrumentation of the cervico-thoracic junction, the use of robust methods such as the pedicle screw is necessary (20), ultimately involving an increased risk of neurovascular complications (11,12), whereby the emphasis here is on the risk of injury, not the incidence of occurrence. Soliman *et al* (13,14) clearly demonstrated that the incidence of vascular injury in pedicle screw fixation can be lowered by navigation. In practice, most spine surgeons will refrain from inserting pedicle screws in the SCS without navigation, with C7 however, being an exception. The results presented in the study by Soliman *et al* (13,14) support the findings of the present study, in confirming the need for a stronger means of fixation than the LMS for complex cases with superior biomechanical characteristics. The screws in our study were placed using the freehand technique, as our center, the Department of Neurosurgery from the Saarland University Medical Center does not routinely implement navigation for screw placement. Nevertheless, navigation is definitely of assistance and should be used when available.

### Screw caliber

As aforementioned, the results presented herein indicate strong similarities between the pedicle and the Attallah screw at this SCS level, making the latter an interesting and safer alternative, whereby lesion morphology always needs to be considered individually. The application of screw lengths and diameters similar to those routinely used in the operating room was a deliberate choice. Specifically, 3.5 mm diameter screws were used for Attallah and LMS, and 4.0 mm diameter screws were used for the pedicle screws. While acknowledging that this may elevate the insertion torque for pedicle screws in contrast to the other screw types, thus favorizing pedicle screws, the primary objective of the present study was to generate results that could furnish valuable data for spine surgeons engaged in real-world clinical practice. Standardizing the diameters could be useful for research purposes, but would have failed in reflecting real-world scenarios.

In conclusion, the results of the present study indicate the biomechanical advantages of the Attallah screw compared to the LMS. However, further research with higher case numbers is necessary to confirm these results. The present study showed relevant subaxial spine level-related variations in the biomechanical properties of the different screw insertion techniques. This is a useful finding that can guide surgical planning. A limitation of the present study was that the three different screws were placed either on the right or the left side, although this is unlikely to restrict its impact. The results gained from cadaveric tissue are noteworthy and indicative but cannot reflect the circumstances in patients. Clinical trials will enable a better understanding of the *in vivo* relevance of the differences observed herein. The ultimate aim is to address the question of whether the combination of safe trajectory and robust screws can lead to a more stable means of fixation of the SCS, with fewer adverse effects.

## Figures and Tables

**Table I:** Diameter and length of the pedicle, Attallah and lateral mass screws.

Level	Pedicle screw	Attallah screw	Lateral mass screw
C3	4.0x24 mm	3.5x20 mm	3.5x16 mm
C4	4.0x24 mm	3.5x18 mm	3.5x12 mm
C5	4.0x24 mm	3.5x16 mm	3.5x12 mm
C6	4.0x24 mm	3.5x16 mm	3.5x12 mm
C7	4.0x24 mm	3.5x20 mm	3.5x10 mm

**Table II:** Cranial and lateral angulation of the pedicle, Attallah, and lateral mass screws.

	Pedicle screw		Attallah screw		Lateral mass screw	
Level	Cranial angulation	Lateral angulation	Cranial angulation	Lateral angulation	Cranial angulation	Lateral angulation
C3	17.5±4.2	40.8±3.8	28.5±7.2	40.8±7.0	27.1±7.0	30.0±0.0
C4	15.0±7.6	41.4±3.8	26.7±9.0	41.3±6.7	28.8±4.4	30.0±0.0
C5	12.9±7.6	40.7±4.5	25.7±8.8	40.7±7.7	29.4±4.2	30.0±0.0
C6	10.0±8.2	41.4±3.8	23.3±11.1	41.0±6.6	29.4±4.2	30.0±0.0
C7	3.6±9.0	40.0±5.8	35.7±6.8	44.0±6.3	27.5±6.0	30.6±1.8

## Data Availability

The datasets used and/or analyzed during the current study are available from the corresponding author on reasonable request.
